# Effectiveness of eHealth cardiac rehabilitation on health outcomes of coronary heart disease patients: a randomized controlled trial protocol

**DOI:** 10.1186/s12872-019-1262-5

**Published:** 2019-11-29

**Authors:** Jing Jing Su, Doris Sau Fung Yu

**Affiliations:** Faculty of Medicine, The Nethersole School of Nursing, the Chinese University of Hong Kong, Room 601, Esther Lee building, Shatin, 999077 Hong Kong

**Keywords:** eHealth, Cardiac rehabilitation, Coronary heart disease, Empowerment, Health behavior

## Abstract

**Background:**

Cardiac rehabilitation (CR) uptake and adherence remain sub-optimal despite the apparent health benefits of modifying healthy behavior and slowing disease progression. eHealth is the use of information and communication technology (ICT) for health. eHealth lifestyle interventions and disease management have emerged as modalities to enhance CR accessibility, enable an individualized progress page, and enrich real-time contact, video-based information, and technology monitored functionality. This study aims to develop a nurse-led eHealth cardiac rehabilitation (NeCR) intervention and investigate its effectiveness on coronary heart disease (CHD) patients’ health outcomes.

**Methods:**

This single-blinded two-arm parallel randomized controlled trial will randomize 146 patients from the inpatient cardiovascular units of a hospital in Wuhan, China to receive either the NeCR or the usual care. The NeCR intervention uses a hybrid approach consisting of a brief face-to-face preparatory phase and an empowerment phase delivered by health technology. The preparatory phase aims at identifying self-care needs, developing a goal-oriented patient centered action plan, incorporating a peer support network and orientation to the use of the e-platform. The empowerment phase includes use of the multi-media interactive NeCR for promoting symptom management, monitoring lifestyle changes and offering psychological support. A tele-care platform is also integrated to enhance health care dialogue with health professionals and peer groups. The control group will receive the usual care. An evaluation of lifestyle behavioral changes, self-efficacy, health-related quality of life, anxiety and depression, cardiovascular risk parameters, and unplanned health services use will be conducted at baseline, 6 weeks and 12 weeks post-intervention.

**Discussion:**

This protocol proposes an individualized, comprehensive, and interactive NeCR delivered using a hybrid approach and guided by an empowerment model to optimize health outcomes of CHD patients. The intervention content and web-design is based on international health guidelines to improve credibility, comprehensibility and implementation. This study also proposes a new method of peer support in which the researcher shares participants’ progress toward goal attainment with the peer group. Results of this research have the potential to increase accessibility and availability of CR, improve cardiac rehabilitation service development in China, and inform eHealth lifestyle interventions.

**Trial registration:**

Chinese Clinical Trial Registry: ChiCTR1800020411; Date of registration: December 28, 2018.

## Background

Coronary heart disease (CHD) is the accumulation of plaque in coronary arteries that limits blood perfusion of the heart [1]. It is related to cumulative exposure to risk factors as well as aging such that 40-year old men and women have a 49 and 32% lifetime risk of developing CHD, respectively [2, 3]. An epidemiologic study indicated that patients with CHD had higher exposure to unhealthy lifestyle behaviors, and physiological and psychosocial risk factors [4]. Using effective behavioral change interventions to address these risk factors could prevent 75% of cardiac events and slow disease progression to heart failure or even death [5]. Current health statistics report 7.4 million deaths globally due to CHD and predict CHD will be the leading cause of disability by 2020 [6].

International guidelines strongly recommend cardiac rehabilitation (CR) programs to manage modifiable risk factors and improve health outcomes of CHD patients. The core components of CR incorporate physical exercise, patient education on lifestyle changes, disease management and psychosocial support [1, 7]. Indeed, CR is a structured program not only addressing direct CHD management but also other conditions including hypertension, dyslipidemia, diabetes and obesity. Substantial benefits associated with CR include reducing mortality by 20 to 47%, reducing hospital re-admission by 18%, improving physical activity, reducing cardiovascular risk factors and improving quality of life [1, 8–10].

Despite the apparent health benefits, 62% of countries do not offer CR, due to non-compatible health and social policy, inadequate infrastructure and manpower [11]. Rate of participation in and adherence to CR is also low and ranges from 14 to 35.5%, due to inadequate access and time conflicts with other life activities [12, 13].

eHealth CR refers to the delivery of CR through information and communication technology (ICT), which has evolved as an alternative modality to improve availability and accessibility of CR. This mode includes a website, mobile applications, monitoring sensors, email, phone calls and short message services [14]. These make eHealth CR unique in being able to promote health behaviors in real-time so that participants can access information, upload self-monitored health data, receive automated feedback, and connect with peers or healthcare professionals [15]. eHealth CR also has the potential of being individually tailored to accommodate individual risk factors, goals and progresses using personal webpage for health behavior change [16]. It also enables experimental teaching that provides video-based health information to facilitate skill building. This modality is based on the statistic that 55.1% of the world’s total population are Internet users [17]. Increasing participatory Internet use has been reported among older adults and become an important health information source and patient empowerment medium for health decision making and behaviors [18]. For this reason, there is increased advocacy to integrate eHealth platforms to deliver care components of CR to address the resource deficiency in traditional CR and provide more accessible and individualized health care to cardiac patients to improve health outcomes.

Existing evidence indicates high feasibility and acceptability of the e-platform to deliver CR [14, 15]. A systematic review and meta-analysis was conducted to examine the effectiveness of eHealth CR on CHD patients [19]. Among the 14 studies reviewed [14, 20–32], meta-analysis indicated that eHealth CR led to significant improvement in physical activity, quality of life and re-hospitalization. There were inconclusive effects on diet, smoking, anxiety and depression, and other physiological risk parameters. This may be due to a lack of emphasis on promoting stress management, smoking cessation and symptom management among the study participants. As compared with studies which reported positive therapeutic effects on health behavioral outcomes, the studies which reported no positive effects were also less likely to incorporate the core strategies of patient empowerment to facilitate health behavioral changes. Such strategies are based on social cognitive theory and refer to individualized cardiovascular risk assessment, goal attainment process and enhanced self-directed tele-monitoring with professional feedback [19–24]. This review provides important insights on how to develop a more promising eHealth CR to enhance behavioral, psychological, physiological and clinical outcomes. All the identified studies in the review were conducted in developed countries including Australia [20], Belgium [21], Canada [22, 23], New Zealand [24, 25], Norway [26], Netherlands [27], Sweden [28], United Kingdom [29, 30], and the United States [14, 31, 32]. None were conducted in Asia/ China, where the cultural difference in health and illness behaviors and relationships with the health care system may limit the generalizability of the findings. China has 772 million Internet users with a penetration rate of 55.8 and 73% nationally and in urban areas, respectively [33]. The ability of older Chinese adults to use an eHealth intervention depends largely on a friendly webpage design, perceived usefulness and culture-compatible components [34, 35]. Therefore, a randomized controlled trial is needed to investigate the effects of an individualized, empowerment-based, and culturally adjusted eHealth CR program to improve health outcomes for people with CHD.

### Social cognitive theory and patient empowerment

The design of this eHealth CR is underpinned by social cognitive theory and patient empowerment model to enhance the translation of online input to actual health behavioral changes [36, 37]. This model uses a patient-centered collaborative approach that patients’ perspectives, ability and resources will be assessed and integrated into formulating self-care goals and action plan. Interactive teaching and experimental learning will be used to enhance patients’ knowledge and skills acquisition as well as transferring the knowledge and skills into behavior change. Self-regulation is also important as patients use proximal and distal goals to guide their behavior and monitor their health behavior and the circumstances under which it occurs.

### Aims

This aims of the study are to develop a nurse-led eHealth cardiac rehabilitation (NeCR) intervention and investigate its effects on lifestyle behavior, self-efficacy, health-related quality of life, anxiety and depression, cardiovascular risk parameters and unplanned health services use.

## Methods

The study design is a single-blinded two-arm parallel randomized controlled trial comparing the effects of an eHealth CR with usual care on the behavioral and clinical outcomes of patients with CHD who are admitted for disease exacerbation.

### Study sample

The study will be conducted at four cardiology units of Tongji Hospital, Wuhan, China where CHD patients are treated conservatively with percutaneous coronary intervention (PCI) or medication and discharged under stable medication regimen. Eligibility requirements of participants are: adults aged 18 years or older; hospitalized for an initial diagnosis of CHD based on angiography or exacerbation of this condition in previously diagnosed cases; anticipated discharge to home; uses a computer and/or smartphone to access the Internet at home, reads and speaks Mandarin; minimum of primary education level; and no prescribed activity or exercise restriction. Exclusion criteria are: a diagnosis of acute psychotic disease/ life-limiting condition; absolute and relative contradictions to exercise testing and training and high risk for exercise prescription according to the American Association of Cardiovascular and Pulmonary Rehabilitation (AACVPR) guideline; and, having auditory, visual, fine motor, or ambulatory disorders.

Referring to a systematic review of studies which examined the effects of tele-cardiac rehabilitation on lifestyle behaviors, health outcomes and quality of life, the effects size on promoting healthy lifestyle is 0.42 to 1.29 so that a medium effect size of 0.5 (with 80% power and .05 significance) is conservatively adopted in this study [38]. The estimated sample size is 146, which takes into consideration an expected attrition rate of 12% based on prior studies with similar post-test time points of 6–12 weeks [31, 39]. Eligible participants will be identified by research nurses from four inpatient cardiac departments of the hospital by reviewing medical records. A second confirmation will be made with the on-site physicians. Eligible participants will then be invited to participate in this study and an information sheet will be provided. After obtaining informed consent, baseline data will be collected.

### Ethics approval

Ethical approval for the study was received from the Joint Chinese University of Hong Kong – New Territories East Cluster Clinical Research Ethics Committee (2018.469). Approval was also obtained from the hospital. The study will be compliant with the Declaration of Helsinki. Protocol modifications will be communicated to the ethics committees.

### The nurse-led eHealth cardiac rehabilitation program

The NeCR program is a health technology based CR program which aims to optimize positive behavioral changes and health outcomes of patients who are discharged following a CHD event. The intervention will be delivered by a cardiac nurse who has extensive experience in clinical teaching and patient education, using a hybrid approach in two phases: i) a brief face-to-face preparatory phase; and, ii) the CR program delivered by health technology using an empowerment approach. Since CR uses a multidisciplinary approach, cardiologists, cardiac nurse, physiotherapist and dietitian were involved in designing the content of the eHealth CR program. A nurse-led approach is used as the cardiac nurse plays a prominent role in supporting patient empowerment and health behavioral changes [40]. Nurses are effective in coordinating care with other health disciplines in rehabilitative services [41]. In implementing the eHealth CR program, the nurse researcher will coordinate the care and seek consultation with the involved disciplines if necessary to optimize the participants’ outcomes. The intervention content is based on updated international guidelines and culturally appropriate national recommendations for CR [42–49].

### Face-to-face preparatory phase

The preparatory phase will be conducted by the nurse researcher prior to the participant’s discharge. It consists of two sessions, with the first session being an individualized health counseling session and the second as a group-based engagement session.

The aim of the individualized health counseling is to identify patients’ self-care needs through a health assessment, develop client-centered goals and a corresponding action plan for lifestyle modification and disease management. The assessment focuses on self-care behaviors for managing CHD including exercise pattern, dietary habits, stress management and tobacco cessation as well as social aspects of disease management such as demographic background, role demand and social support to identify self-care needs. Patients’ function capacity will be assessed by submaximal test using remote electrocardiography (Mindray Tel-200, China) monitored six-minute walk test, based on which the metabolic equivalent (MET) will be calculated by the nurse [46, 50]. Accordingly, the nurse will provide brief health counseling based on the international guidelines recommendations for CR. The discrepancy between the patients’ self-reported behaviors and the guideline recommendation will be highlighted, and the impact of this on health outcomes will be elaborated upon. By increasing the participants’ awareness of their own self-care deficits, the nurse will support them in developing their goals for improving exercise patterns, dietary behaviors, and stress management.

The goal setting for exercise will indicate the frequency, duration, and intensity of exercise to gradually achieve at least 150 min of moderate weekly exercise. The nurse will also teach the participants about using Borg’s ratings of perceived exertion to achieve the desired intensity with a perceived exertion of 12 to 14 (“somewhat hard” on a scale of 6 to 20) [51]. For dietary habits, a guideline based culturally appropriate 10-item dietary habit checklist has been developed assessing food intake, cooking methods, eating pattern, and method of eating in restaurants. This checklist is adapted from the dietary checklist designed by the Department of Health in Taiwan to guide the dietary modification for cardiac patients [52]. The research team has modified the content so as to make it culturally relevant to the CHD patients in China. The revised content has been validated by two Chinese dietitians. Participants will be supported in setting successive goals to eventually attain over 90% adherence to this checklist. As for stress management, participants will be supported to set goals of managing stress below a level of 3 as assessed by 0–9 scored scale [53]. The nurse will collaborate with the patients to work out the action plan with a variety of exercise, dietary and relaxation skills options. Participants who smoke will also be supported to set a cessation goal and provided with tips such as nicotine replacement therapy, avoid smoking for social influences and managing stress [54]. A written participant-centered goal-driven action plan and information handout for self-management will be provided to reinforce knowledge retention. The individualized goals and action plan will be uploaded to the participants’ password protected personal webpages on the eHealth CR platform.

The group-based engagement session will take place in the hospital meeting room with 4~6 participants per group. The aims are: i) orientate participants to the operation of the web- and tele- platform, and ii) form a cohesive peer support group to optimize lifestyle behavioral changes. The nurse will share anonymously the goals of the participants and discuss the importance of goal attainment in optimizing health outcome. The nurse will give an orientation to the web-platform by demonstrating each key feature and distribute the e-platform user manual. In order to ensure the success of the subsequent self-monitoring on physical exercise, a pedometer with written instructions, will be provided. Practice and return demonstration is needed to ensure skill mastery. Finally, the nurse will invite participants to enter a WeChat telecare platform consisting of 12 ~ 16 participant members.

#### CR program delivery by health technology using an empowerment approach

The 12-week CR program is the core component of the intervention and aims at using two core methods including an interactive and motivational web platform, and a tele-care platform to optimize CR participation and behavioral modification.

The interactive and motivational web-based platform consists of three key platforms: a) self- monitoring with motivational feedback platform, b) an interactive and experiential learning platform, and c) health dialogue forum. The design of this platform followed the guideline of the Health Literacy Online to improve the comprehensiveness and utility of the content [55]. The content was revised to be at the readability level of sixth to ninth grade examined by Microsoft® Word® Office Package (Microsoft Corp, Redmond, WA) to optimize comprehension. The web platform can be automatically adjusted to computer and smartphone interfaces to improve flexibility and access.

#### Self- monitoring with motivational feedback platform

The self-monitoring page will document the goals and action plans developed for improving exercise level, dietary habits, stress management and/ or tobacco cessation during the preparatory phase. Participants are recommended to input self-monitoring data on a daily/weekly basis to indicate the level of goal attainment. For exercise, participants will upload exercise times, the intensity level as light/moderate/vigorous and the duration of engagement in 1 week. In addition, the guidelines for CR also recommend a higher level of walking to exceed 7000 steps [56], and each participant will be encouraged to upload their daily step count measured by their pedometer. Participants will be encouraged to complete the guideline based dietary habit checklist and their score on the 0 to 9 stress scale to indicate their dietary habit and stress level. A traffic light system will be used to give motivational feedback based on the level of goal attainment, with red indicating attention required (< 50% of goal attainment), yellow representing moderate achievement (> 50–90% of goal attainment) and green indicating full attainment (90–100% of goal attainment). An automated message which corresponds to the level of goal attainment and shares the trend in participants’ recordings will be presented. Patients will be asked to report on tobacco use and encouraged to update their smoking status (if applicable) with green indicating they have quit while red represents smoking. Based on the pre-determined coding, an overall score on goal attainment will be generated along with the traffic light system to reflect progress on each behavioral change.

#### Interactive and experiential learning platform

The interactive and experiential learning platform provides all intervention group participants access to comprehensive health information regarding CHD. The content was developed according to the updated international guidelines and culturally appropriate national recommendations for CR by the American Heart Association, American Psychological Association, American Diabetes Association, The American Cancer Society, Chinese Nutrition Society, and the National Center for Cardiovascular Disease [42–49]. The content covers the pathophysiology and manifestation of CHD, physical activity, diet management, smoking cessation, stress management, risk factors management (hypertension, cholesterol, and diabetes), symptom management and post-PCI management. The health information has been adapted to fit in the Chinese context, and the content of this platform has been validated by a cardiologist, dietitian and cardiac nurses. To motivate participants, each component is presented sequentially: i) introducing the role and underlying mechanism of each care component in disease management, ii) the required lifestyle changes, iii) recommended actions, and iv) self-monitoring to track progress and resolutions to barriers. Various learning opportunities will be provided using scenario-based case sharing to illustrate behavioral change barriers, challenges and resolution alternatives; videos for skills demonstrations and role modeling; and pictures, slogans and comics to present these sessions in an engaging manner. The health dialogue forum aims at providing a method for posting CR research news and answering questions from participants.

#### Tele-care platform

In the tele-care platform, the nurse will retrieve self-monitoring data uploaded by participants and anonymously share goal attainment of group members as well as discuss their experiences and address any concerns. Reminders for uploading data will be sent to participants weekly. This platform permits participants to contact the nurse about issues or adverse changes in health. The nurse will contact each individual at weeks 2 and 4 post-discharge to assess fitness, explore difficulties, encourage progress and offer solutions. This may lead to revision of the action plan or goal adjustment. Participants will be asked to maintain password and account security, and to avoid discussing of intervention content with other patients in the hospital to avoid contamination.

### Usual care

Participants in the both groups will receive the usual health care information related to maintaining a healthy lifestyle (exercise, diet and smoking cessation), instructions on medication during hospitalization, and one follow-up call. The research nurse will instruct control group participants on use of the pedometer.

### Randomization and data collection procedure

**Fig. 1:**
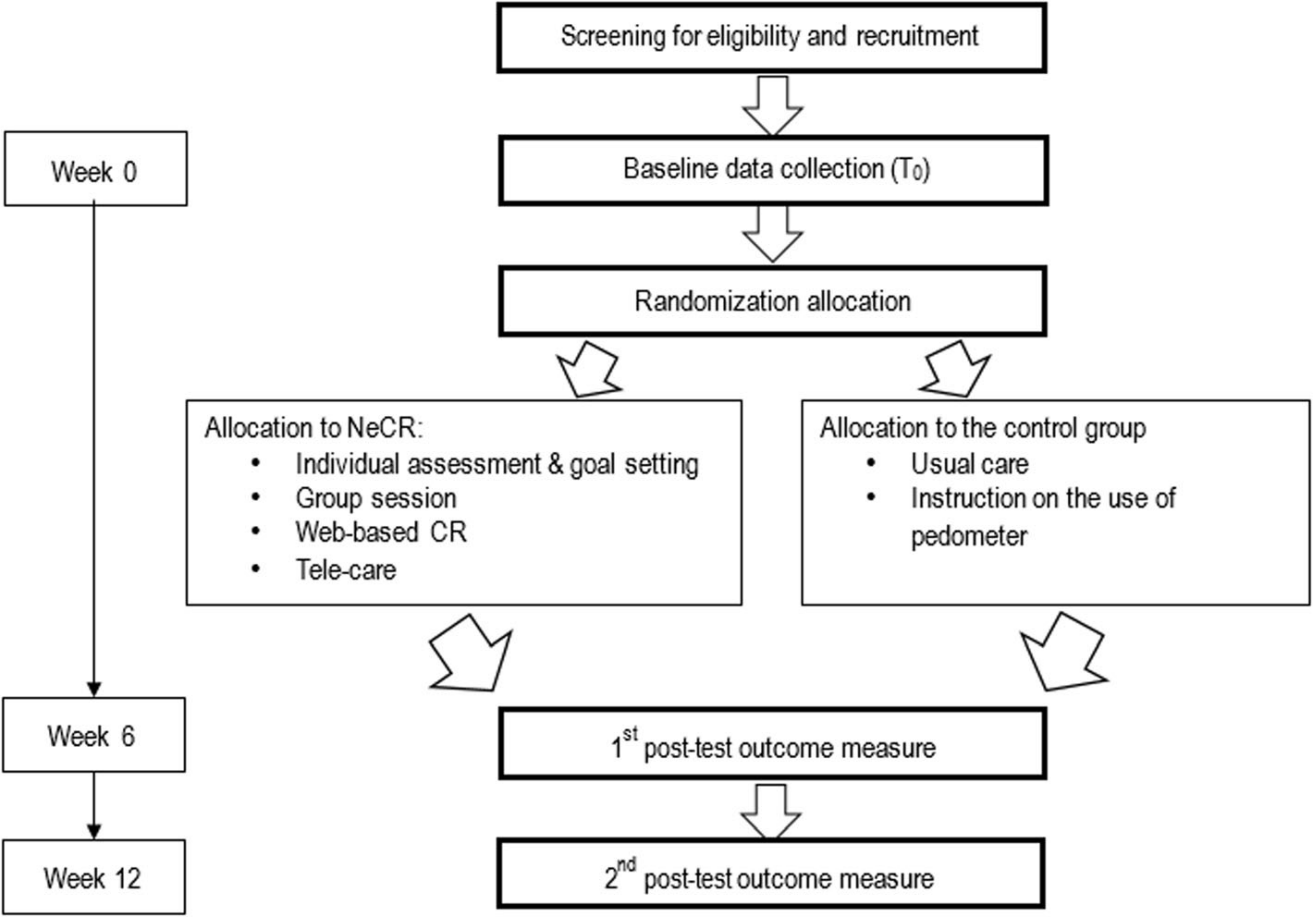
Flow diagram of the study protocol

Eligible patients will be invited to participate in this study and an information sheet will be provided. After obtaining informed consent, baseline data will be collected. Participants will be randomly assigned to either the intervention or control group by block randomization. Three different block sizes of 4, 6 and 8 will be used. The random number for group assignment will be generated by random allocation software. The group assignment will be written and placed in an opaque sealed envelope and given to participants by the research assistant after baseline data collection (T_0_). Outcome evaluation will take place at the 6-week intervention (T_1_) and upon completion of the 12-week intervention (T_2_) by trained research assistants who are blinded as to the participants’ intervention allocation (Fig. 1). Participants will not be blinded as to their group allocation. The following parameters for outcome assessment will be used to include health behavior, emotional outcomes, health-related quality of life, cardiac physiological risk parameters and unplanned health services use. Participants’ travel expenses for data collection will be reimbursed.

### Primary outcomes

The primary outcomes focus on health behavioral change including physical activity, health promoting lifestyle habits, and smoking behaviors. Physical activity is defined as movement of the body that uses energy. Objectively, physical activity level will be measured by the pedometer (Mi Band, China) record of step count for three weekdays [57]. A pedometer correlates strongly with different accelerometers (median r = 0.86) as well as the time in observed activity (median r = 0.82) [58]. The International Physical Activity Questionnaire (IPAQ) will be used to comprehensively assess the self-reported time spent in walking, light/moderate/rigorous exercise and sitting across various domains using 27 items. The IPAQ has test–retest reliability with intra-class correlation coefficients of 0·74 to 0·97 for each sub-domain for Chinese adults [59]. Total physical activity measured by the IPAQ was moderately correlated with pedometer measured exercise levels (r = 0.46) [60].

Health promoting behaviors will be measured by the Health Promoting Lifestyle Profile II (HPLP- II) [61]. The HPLP- II measures six aspects of health promotion behaviors including nutrition, interpersonal support, stress management, exercise, health responsibility and self-actualization. The HPLP - II has good internal consistency with 0.92 for the entire scale and 0.69–0.84. The HPLP uses a four-point scale with 1–4 representing always to never with a total score ranging from 40 to 160 and a higher score indicating better health behavior. The HPLP-II has been translated and validated into Chinese with Cronbach’s α coefficient of 0.63 to 0.81 [62].

Smoking status will be asked to evaluate the abstinence rate using two questions: (1) what is your current smoking status? (current smoker, ex-smoker or nonsmoker) (2) How many cigarettes do you smoke per day? [63].

### Secondary outcomes

Cardiac self-efficacy refers to an individual’s confidence in managing cardiovascular disease. It will be measured using the Cardiac Self-efficacy Scale (CSES), originally developed by Sullivan [64]. The 13 item questionnaire consists of two subscales to measure one’s self-efficacy in maintaining function and to control symptoms. The CSES uses a 5-point Likert scale with a higher score indicate higher self-efficacy. The original version of the CSES has good internal consistency with Cronbach’s alpha of 0.90 and 0.87 for the subscales. The CSES has been translated and validated among Chinese cardiac patients with internal consistency of 0.926. The construct validity of the C-CSES was established with significantly moderate correlations General Self-efficacy Scale (r = 0.47, *p* < 0.001) [65].

Health-related quality of life will be measured by the MacNew Heart Disease health related quality of life questionnaire (HRQoL), which evaluates how an individual’s emotional, physical and social well-being are affected by CHD and its treatment. It has 27 items scored from 1 (low HRQoL) to 7 (high HRQoL) with internal consistency and intraclass correlation coefficients≥0.73 [66]. It has been translated and validated among Chinese CHD populations with intraclass correlation coefficient ranging from 0.88–0.93 [67].

Psychological status of participants will be measured by the Depression Anxiety Stress Scale 21 (DASS-21) consisting of 21 items in three domains: depression, anxiety and stress [68]. Respondents are required to indicate the presence of the symptom(s) over the past week on a 4-point Likert scale scoring from 0 to 3 with higher scores indicate more severe symptoms. This instrument has well-established psychometric properties in reliably measuring depression, anxiety and stress (Cronbach’s alpha of 0.91, 0.84 and 0.90 respectively). The DASS-21 correlated strongly with hospital anxiety and depression scale both for anxiety (r = 0.88) and depression (r = 0.93) [69]. The DASS-21 has been translated and validated among Chinese with Cronbach’s alpha≥0.80 [70, 71].

The cardiac physiological risk parameters include body mass index (BMI), blood pressure and waist circumference. Body weight and height will be examined using the same equipment (Xiheng, RGZ-120-RT, China) with participants in light clothing, shoes removed, upright posture and looking straight ahead. Blood pressure will be measured in a sitting position after participants have rested for 10 min (Omron HEM-7124, Japan). Waist circumference will be measured using a flexible tape measure at umbilicus level.

The unplanned health services use is defined as unplanned cardiac-related hospital readmissions, emergency department visits and re-vascularization. Because China does not have an electronic medical records system, the participants will be reminded to call the outcome assessor whenever an unplanned health services event occurs. To reduce recall bias, the outcome assessor will assess participants’ unplanned medical services use by monthly call asking about the detailed date, unit, diagnosis and related information. Survival analysis of time-to-event data will be conducted to describe the length of time from entering the program to any readmission.

### Demographic and clinical data

Comprehensive demographic and clinical date will be collected at baseline to permit examination of factors that may influence the NeCR outcomes of participants. Demographic data (age, sex, education, marital status, employment status, living conditions and Internet use) will be collected from participants while clinical data will be retrieved from the medical record to include diagnosis, clinical presentation, documented hypertension, dyslipidemia, diabetes, any treatments being received and single/multiple vessel disease.

### Data management

Participants will be given a code to ensure anonymity. According to Chinese University of Hong Kong Policy on Research, Intellectual Property and Knowledge Transfer, data will be stored in a locked computer accessible only to the principal investigator and researcher [72]. Data entry of all questionnaires will be validated by a second person.

### Data analysis

The latest version of SPSS will be used to analyze data with a 5% significance level (two-sided). Treatment evaluation will be performed based on the principle of intention to treat. A generalized estimating equations (GEE) model will be used to compare the differential between the two groups related to each of the outcomes across the time-points, with adjustment for potential confounding variables in order to obtain a more precise estimation of intervention effect. The potential confounding variables will be selected on the basis of clinical judgment and statistical incomparability at baseline. For the smoking behavior as nominal data, the Chi-square test will be used to compare the number of patients who quit smoking between the groups. In addition, the Friedman test will be used to compare the changes in percentage of smoking cessation among the groups over time [73]. Survival analysis, the analysis of time-to-event data will be conducted to describe the length of time from entering the program to an unplanned health services utilization.

### Process evaluation

Experiences and perceptions of participants in the NeCR will be assessed through individual in-depth interview of a sample of 15–20 intervention group participants. Sampling will occur until theoretical saturation, which is expected to be reached by selection of 20% of the intervention participants [74, 75]. To maximize the sample variation, participants who have different responses to the NeCR in terms of the changes on their scores on the primary outcomes (0-35th percentile, >35th percentile and > 70% percentile of the score) will be recruited. The subject selection will also attempt to optimize the relevant clinical and demographic characteristics such as age, gender, education and treatment received. Interviews will be conducted by a researcher with extensive experiences in qualitative interviewing and independent of outcome assessment. The interview will focus on individual NeCR experiences, perceived effectiveness, likes and dislikes and further suggestions. Thematic content analysis will be used including an iterative process reading, coding, categorizing and identifying themes [76].

Engagement with the NeCR will be assessed using the website log file for numbers of log in and data upload. Numbers of communication with the nurse for consultation and events or issues sharing among peers will be recorded.

### Potential risks management

All personal electronic data will be stored in a password-protected account to ensure confidentiality. Participants have been informed that they will incur no additional costs for care received during the intervention and it will not replace care provided by their physician. Participation in the study will be discontinued if a participant’s health status changes and no longer meets inclusion criteria and they will be referred to appropriate care services. To minimize any possible adverse event due to exercise, patients will be advised to start exercise at their metabolic equivalent level based on a six-minute walk test and increase gradually in frequency, duration, and intensity. Participants will be encouraged to access symptom management information and contact the nurse for guidance when needed. An emergency card with participant’s name, diagnosis, contact information of the family/friend and researcher will be provided in case of an adverse event due to disease deterioration or exercise.

## Discussion

This study will use a hybrid approach guided by an empowerment model to investigate the effect of eHealth CR which integrates behavior change strategies on not only exercise but also on diet, stress and smoking. The intervention content and web-design are based on international guidelines and national culturally appropriate recommendations to improve credibility, comprehensibility and implementation for this population. Individual assessment and a collaborative approach to goal setting and action planning helps participants understand the purpose of using the web- and tele-platform. This could also encourage participants’ engagement in self-care decision making with their health care professional and proactive use of the e-platform, which are resources for self-management. Considering the continuous care needs of a patient discharged to their home setting after a cardiac event, individual consultation and guidance from a nurse and encouragement from peer interaction and support will be initiated through multi-communication channels as social motivators for behavioral goal attainment. This study uses a new method of peer support in which the researcher shares participants’ progress toward goal attainment with the peer group and encourages participants to share their experiences and concerns to reinforce behavioral changes.

### Limitations of the study

There are several possible limitations to this protocol. Although participants will be recruited from a regional hospital which serves a population with diverse demographic and socio-economic characteristics, the generalizability of the study findings will still be limited since the study involves only one province in China and excludes those residents who are illiterate. Another limitation is that self-report measures are used to monitor the level of participation in the eHealth CR and the changes in behavioral, psychological and health-related status. The findings may subject to social desirability and recall bias. Also, even though an attention placebo in the form of patient education is used in this clinical trial, none of the initiatives can ensure that an equal amount of attention is paid to both groups. This may result in a threat to internal validity.

## Conclusion

This study reports the development and evaluation of a novel eHealth CR program for the Chinese CHD population. By using a hybrid approach to integrate minimal personal contact with intensive and multimodal eHealth initiatives, the eHealth CR program addresses the limited accessibility and acceptability of the conventional CR program without scarifying its merit in providing encounters with healthcare professionals. With the detailed information about the program content and implementation plan as well as the evaluation in the process and outcome perspectives, this paper provides insights to enable the model replication or application in the management of other chronic diseases.

## Data Availability

Datasets from this study will be available from the corresponding author upon reasonable request.

## Abbreviation

AACVPR: American association of cardiovascular and pulmonary rehabilitation
BMI: Body mass index
CHD: Coronary heart disease
CR: Cardiac rehabilitation
CSES: Cardiac self-efficacy scale
DASS-21: Depression anxiety stress scale 21
GEE: Generalized estimating equations
HPLP- II: Health promoting lifestyle profile II
HRQoL: Health related quality of life questionnaire
IPAQ: International physical activity questionnaire
MET: Metabolic equivalent
NeCR: Nurse-led eHealth cardiac rehabilitation
PCI: Percutaneous coronary intervention
